# Quantitative examination of factors influencing the colour reproduction ability of lithium disilicate glass-ceramics

**DOI:** 10.1186/s12903-024-04429-w

**Published:** 2024-06-05

**Authors:** József Saláta, Ferenc Szabó, Péter Csuti, Melinda Antal, Péter Márton, Péter Hermann, Judit Borbély, Emese Ábrám

**Affiliations:** 1 Department of Prosthodontics, Faculty of Dentistry, Semmelweis University, Szentkirályi Street 47, Budapest, H-1088 Hungary; 2 School of PhD Studies, Semmelweis University, Budapest, Hungary; 3 LightingLab Calibration Laboratory Ltd, Veszprém, Hungary; 4 Faculty of Dentistry, Semmelweis University, Budapest, Hungary

**Keywords:** Lithium disilicate, Dental ceramic, Colour, Colour difference, Spectrophotometer, Translucency, Thickness, Substrate, Dental materials, Materials science

## Abstract

**Background:**

Effects of ceramic translucency, layer thickness, and substrate colour on the shade of lithium disilicate glass-ceramic restorations proved to be significant in several studies, however, quantitative, numerical results on the relationship between the colour difference and these parameters are still lacking. The purpose of this in vitro study was to quantitatively determine how the colour reproduction ability of a lithium disilicate glass-ceramic is affected by its translucency, layer thickness, and substrate colour.

**Methods:**

Ceramic samples were prepared from A2 shade IPS e.max CAD blocks with high and low translucencies (HT and LT) in a thickness range of 0.5–2.5 mm (+/- 0.05 mm). Layered samples were acquired utilizing composite substrates in 9 shades; transparent try-in paste was used. The spectral reflectance of the specimens was assessed under D65 standard illumination with a Konica Minolta CM-3720d spectrophotometer. The CIEDE2000 colour difference (ΔE_00_) between two samples was analysed using perceptibility and acceptability thresholds set at 50:50%. Statistical analysis involved linear regression analysis and the Kruskal–Wallis test.

**Results:**

An increase in the thickness of 0.5 mm reduced the ΔE_00_ of the HT samples to 72.8%, and that of the T samples to 71.1% (*p* < 0.0001). 7 substrates with HT and LT specimens had significantly different results from the mean (*p* < 0.05). A thickness of 0.5 mm is not sufficient to achieve an acceptable result at any level of translucency, while the low translucency ceramic at a thickness of 1.5 mm gave acceptable results, except for severely discoloured substrates (ND8 and ND9).

**Conclusions:**

The colour reproduction ability of lithium disilicate glass-ceramics is significantly affected by their translucency, layer thickness, and 7 substrates out of 9 substrates examined.

## Background

There are countless possibilities for the use of dental ceramic materials in the 21st century due to their excellent optical and mechanical properties. The evolution of computer-aided design/computer-aided manufacturing (CAD/CAM) technology, which simplifies machinability, further increased the popularity of these materials [1, 2]. In addition, their structural modifications resulted in an increase in flexural strength, enabling monolithic application of certain silicate ceramics [3, 4]. Today, aesthetic dentistry would be unimaginable without glass-ceramics, which are highly popular monolithic restorative materials due to their outstanding translucency, low thermal conductivity, appropriate mechanical properties, biocompatibility, and wear resistance [4–7]. Although the first dental glass-ceramics appeared on the market in 1984, structural modifications to improve their mechanical properties have not since ended [8, 9]. Among the currently available glass-ceramic materials, lithium disilicate glass-ceramics (LS_2_) are the most resistant (up to 370–460 MPa flexural strength) and the most widely used [5, 10–12]. The first LS_2_ was introduced in 1998 under the name IPS Empress II (Ivoclar Vivadent, Schaan, Liechtenstein) and was processed using the lost-wax technique and pressing [13, 14]. The spread of CAD/CAM technology and chair-side dentistry required the development of a millable LS_2_ material by 2005 (IPS e.max CAD, Ivoclar Vivadent, Schaan, Liechtenstein) [13, 15]. The material is available as a purple, precrystallized block with a low flexural strength of approx. 130 MPa, thus enabling fast milling and less wear on milling tools [12, 16]. After milling, the final colour is acquired during final crystallization, which requires 10 min at 850°C [12, 14]. The flexural strength of 360 MPa thus achieved resulted in the expansion of the indication area of the material: monolithic inlays, onlays, partial and full crowns, and bridges (up to 3 units) can be made of it [4, 17]. The versatile use of IPS e.max CAD is facilitated by the fact that it is available in five different translucencies: high translucency (HT), medium translucency (MT), low translucency (LT), medium opacity (MO), and impulse (I).

The main goal of aesthetic dentistry has always been the most lifelike reproduction of natural teeth and tooth tissues. With ceramic materials available to us today, all possibilities are given to achieve this; however, a very thorough knowledge of the materials and technologies used is necessary for a perfect result [18]. Numerous studies have proven that in the case of indirect restorations, in addition to the shade of lithium disilicate glass-ceramics, the abutment (substrate) and cement under the restoration can have an effect on colour, as well as the translucency and layer thickness of the ceramic [1, 18–32]. Although the colour-modifying effects of the ceramic translucency, layer thickness, and substrate colour proved to be significant in several cases, quantitative, numerical results on the relationship between the colour difference and these parameters are still lacking. Spectrophotometers capable of measuring the light reflected from the surface and deeper layers of an object are suitable for objective examination of the colour of such a complex system. During in vitro tests, if possible, it is preferable to use a laboratory spectrophotometer, which can produce measurement results with industrial accuracy. In this way, the spectral reflectance of an object can be obtained, which may lead to further comparisons. The colour difference (ΔE) calculated from a reference makes the colour appearance and colour reproduction ability of the objects comparable to each other and reveals the effect of changing certain parameters.

The purpose of this in vitro study was to quantitatively determine how the colour reproduction ability of a lithium disilicate glass-ceramic is affected by its translucency, layer thickness, and substrate colour.

The following null hypotheses were tested in the study:

1. The colour reproduction ability of the lithium disilicate glass-ceramic is not significantly affected by the layer thickness of the ceramic.
2. The colour reproduction ability of the lithium disilicate glass-ceramic is not significantly affected by the substrate colour.
3. The colour reproduction ability of the lithium disilicate glass-ceramic is not significantly affected by the translucency of the ceramic.

## Methods

According to a pilot study, to detect a ΔE difference of one standard deviation between two compared groups at 5% significance and 80% power, 17 observations per group, or 34 observations in total, are needed.

### Ceramic specimen preparation

**Fig. 1:**
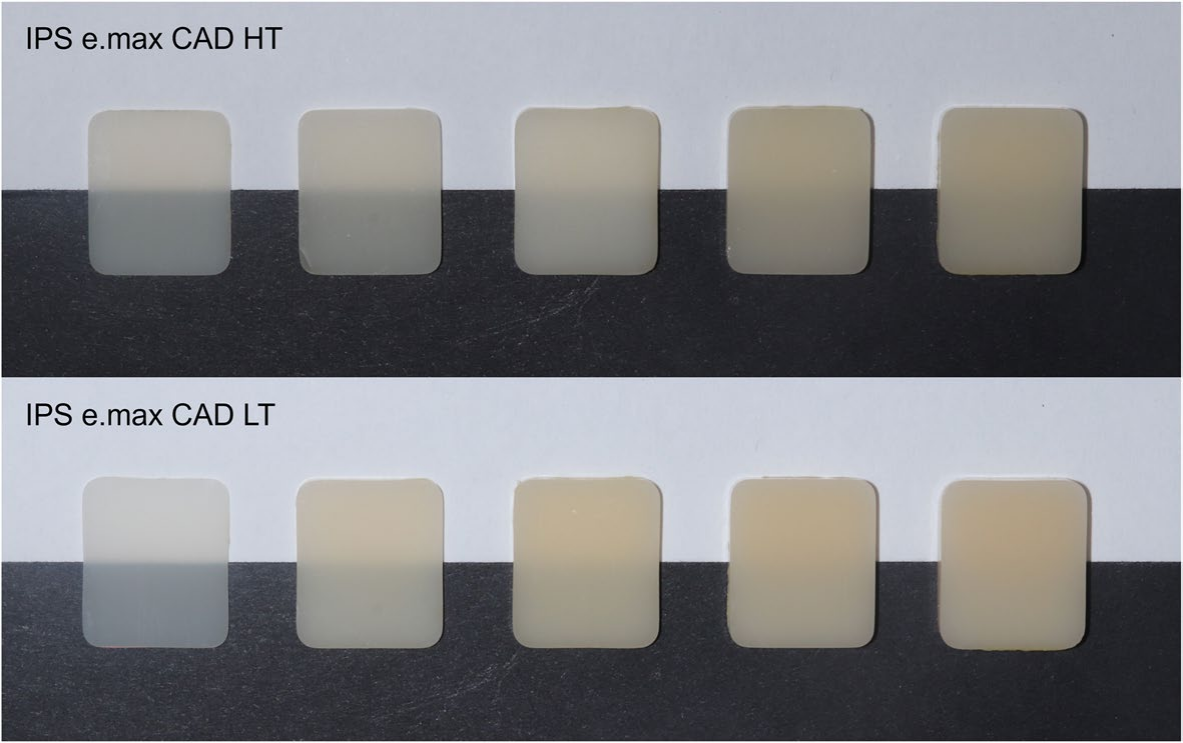
High and low translucency lithium disilicate glass-ceramic specimens with thicknesses of 0.5 mm; 1.0 mm; 1.5 mm; 2.0 mm; and 2.5 mm (from left to right)

Lithium disilicate glass-ceramic specimens were prepared from A2 shade IPS e.max CAD material (Ivoclar Vivadent, Schaan, Liechtenstein) with 2 different translucencies (high translucency ‘HT’ and low translucency ‘LT’) for in vitro examination. First, rectangular ceramic specimens were made of precrystallized blocks with side lengths of 12 mm × 14 mm. The thickness was calculated so that after the subsequent final crystallization and linear shrinkage of 0.2% [17], ceramic specimens with layer thicknesses of 0.5 mm; 1.0 mm; 1.5 mm; 2.0 mm and 2.5 mm (+/- 0.05 mm) were obtained (3 pieces of each thickness, *n* = 30) (Fig. 1). A diamond disc slicer (T-CG-04 01/2016, Tenzi, Budapest, Hungary), a grinding machine (T-CG-05 04/2018, Tenzi, Budapest, Hungary) and SiC800 grinding powder were used to cut and size the precrystallized ceramic slices under continuous water cooling. The final crystallization of the samples was carried out in a furnace (Programat P300, Ivoclar Vivadent, Schaan, Liechtenstein) according to the manufacturer’s instructions. Afterwards, both surfaces of the ceramic specimens were polished with a suspension of 0.5 μm cerium oxide powder and water, using a polishing plate. The thickness of the ceramic slices was validated by a digital micrometer (Mitutoyo, Kawasaki, Japan). As a reference, A2 shade HT and LT IPS e.max CAD blocks were used, which were also crystallized according to the manufacturer’s instructions. The surfaces of the ceramic blocks were polished with a suspension of 0.5 μm cerium oxide powder and water, using a polishing plate.

### Substrate preparation

Substrate materials were also used in the examination to simulate the prepared abutment. Substrates were made of a special light-curing composite (IPS Natural Die Material, Ivoclar Vivadent, Schaan, Liechtenstein) in 9 shades (ND1, ND2, ND3, ND4, ND5, ND6, ND7, ND8, and ND9). Transparent silicone impression material (Exaclear, GC, Tokyo, Japan) was used to make a template of a rectangular cuboid with side lengths of 20 mm × 20 mm × 8 mm. The silicone template was infused with the composite substrate material. Polymerization was conducted using a light polymerization unit (EyeVolution, Dreve ProDiMed, Unna, Germany).

### Assemblage of layered specimens

**Fig. 2:**
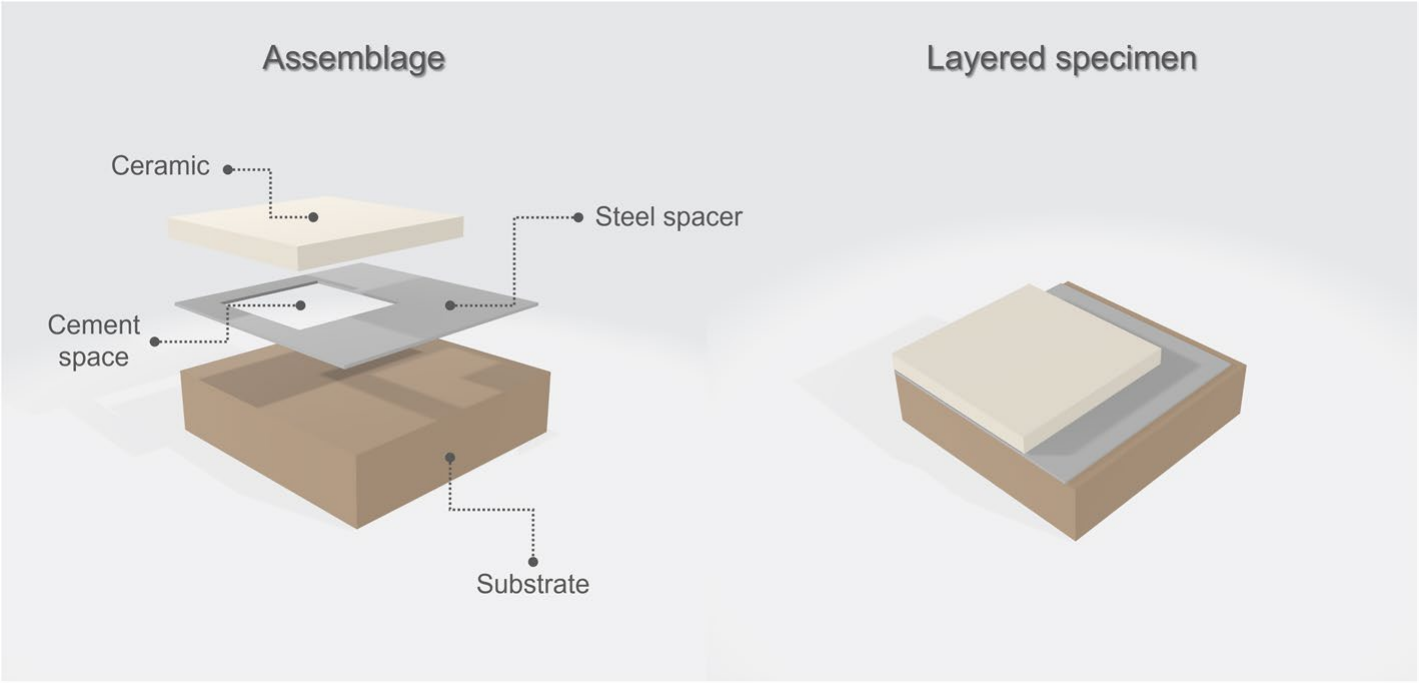
Schematic figure of the assemblage of a layered specimen consisting of a ceramic sample, steel spacer, try-in paste, and substrate

Layered specimens were assembled using ceramic specimens, substrates and transparent try-in paste (Variolink Esthetic Try-In Paste (Neutral), Ivoclar Vivadent, Schaan, Liechtenstein) with a layer thickness of 100 μm. To ensure the standard layer thickness of the try-in paste, a 100 μm thick steel spacer and an automatic pipette were used (Fig. 2). Each ceramic sample was combined with all the substrates; thus, 45 layered specimens per group (HT and LT), 90 layered specimens in total were observed.

### Spectrophotometric measurements

The spectral reflectance of the specimens was measured using a Konica Minolta CM-3720d (Konica Minolta, Tokyo, Japan) spectrophotometer in the wavelength range of 360–740 nm at a 10 nm pitch, with a d/8 (diffuse illumination/8° viewing angle) measurement geometry and specular component included (SCI) setting [33]. The apparatus features a 6-inch integrating sphere coated with barium sulphate, exhibiting superior optical characteristics. From the spectral reflectance, the L*, a*, and b* values were calculated according to the D65 standard illumination. Three measurements were performed without replacement, and the results were averaged.

### Colour difference calculation and statistical methods

To determine the colour reproduction ability of the ceramic specimens, colour differences (ΔE_00_) were calculated between individual layered samples and specific reference samples. The results of the layered specimens containing HT ceramic were compared with the results of the HT ceramic block (as a target colour), and the results of the layered specimens containing LT ceramic were compared with the results of the LT ceramic block. The colour difference (ΔE_00_) between two samples was calculated using the CIEDE2000 formula [34] (valid since 2000):

$${\varDelta E}_{00}^{*}=\sqrt{{\left(\frac{\varDelta L{\prime }}{{k}_{L}{S}_{L}}\right)}^{2}+{\left(\frac{\varDelta C{\prime }}{{k}_{C}{S}_{C}}\right)}^{2}+{\left(\frac{\varDelta H{\prime }}{{k}_{H}{S}_{H}}\right)}^{2}+{R}_{T}\frac{\varDelta C{\prime }}{{k}_{C}{S}_{C}}\frac{\varDelta H{\prime }}{{k}_{H}{S}_{H}} }$$

The formula is recommended by the International Commission on Illumination (Commission Internationale de l’Éclairage, CIE) [35], as it has been demonstrated to offer a superior fit to visual perception compared to CIE76 [36], and the parameters ΔL′, ΔC′, and ΔH′ in the formula represent differences in lightness, chroma, and hue values between two samples. R_T_ denotes the hue rotation term applied to weighted hue and chroma differences. S_L_, S_C_, and S_H_ are weighting factors, and the parametric factors k_L_, k_C_, and k_H_ are correction terms for variation in the experimental conditions [35]. PT_50:50%_ = 0.8 (50:50% perceptibility threshold) and AT_50:50%_ = 1.8 (50:50% acceptability threshold) ΔE_00_ values were utilized to assess the results of colour differences [33, 37, 38]. The Kruskal–Wallis test was used to analyse whether samples had the same distribution (*p* < 0.05). The effects of layer thickness and substrate on the colour reproduction ability were analysed using linear regression (*p* < 0.05). The statistical package Stata (StataCorp LLC, Collage Station, Texas, USA) was used for data handling and analysis.

## Results

**Fig. 3:**
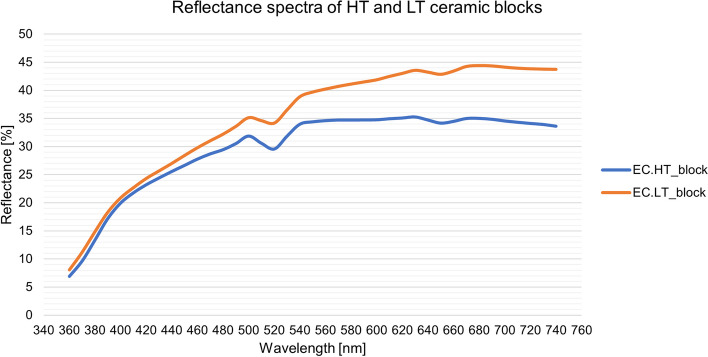
Reflectance spectra of the HT and LT ceramic blocks

A comparison of the reflectance spectra of the HT and LT ceramic blocks used as a reference was carried out to evaluate the basic reflection properties of the two ceramic types (Fig. 3).

**Fig. 4:**
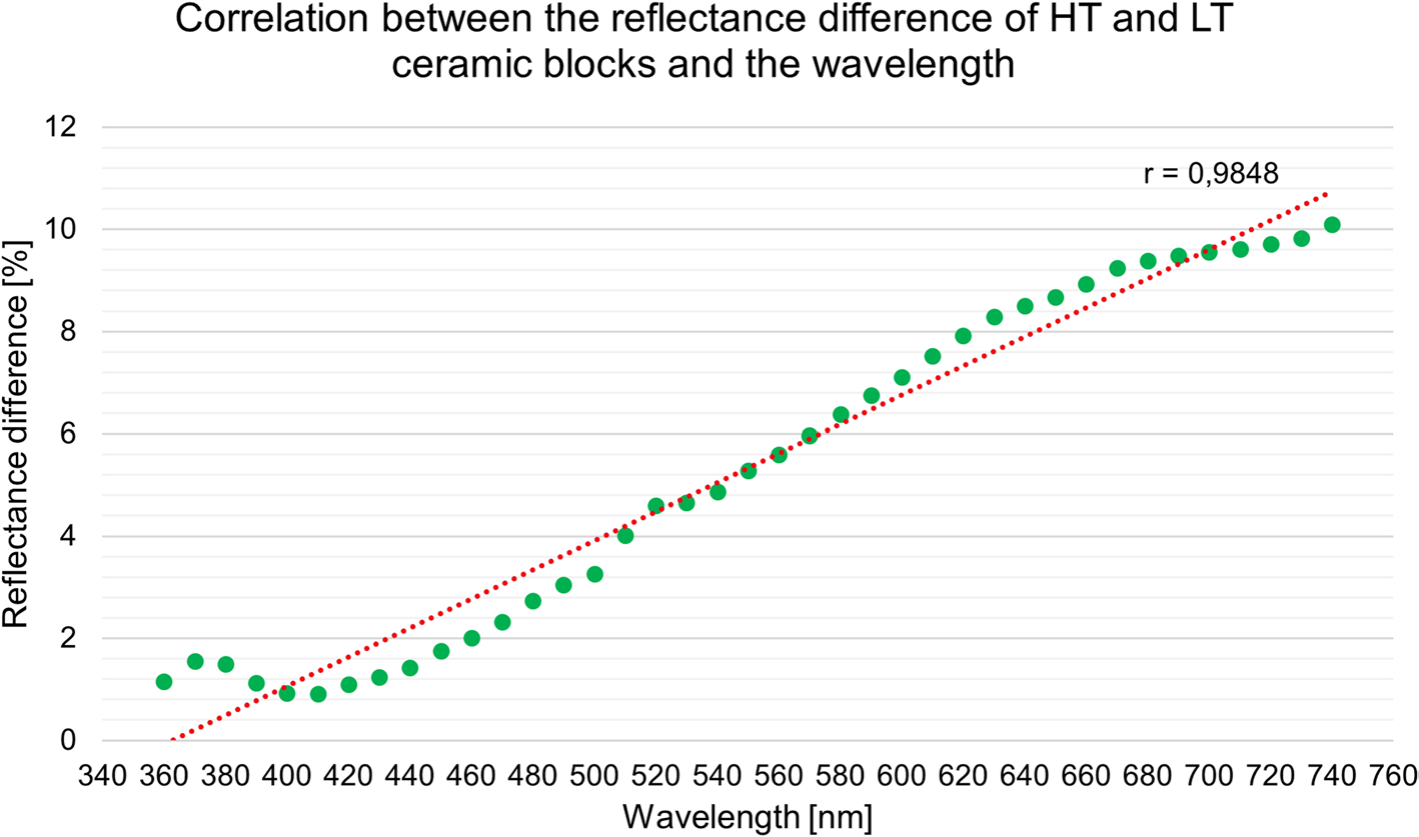
Linear correlation between the difference in the reflectance of the HT and LT ceramic blocks and the wavelength (*r* = 0.9848)

The relationship between the difference between the spectral reflectance of the two materials and the wavelength was analysed using linear regression (Fig. 4). *R* = 0.9848 linear correlation coefficient with *n* = 39 sample size shows a very high level of linear correlation (α = 0.05).

**Fig. 5:**
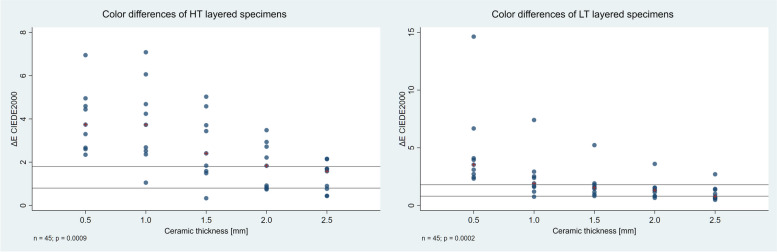
Dependence of ΔE_00_ values of the HT and LT layered specimens on ceramic thickness. Reference samples: HT and LT blocks. The median of each group is indicated by a red cross. PT_50:50%_ = 0.8 and AT_50:50%_ = 1.8 are marked on the diagram

The relationship between the colour difference (ΔE_00_) of the HT and LT layered specimens from the reference and the ceramic layer thickness is shown in Fig. 5. Beside every layer thickness, there is a group of 9 observations corresponding to the measurements with the 9 substrates. The median of each group is indicated by a red cross. Perceptibility (PT_50:50%_ = 0.8) and acceptability (AT_50:50%_ = 1.8) thresholds are marked by horizontal lines.

**Fig. 6:**
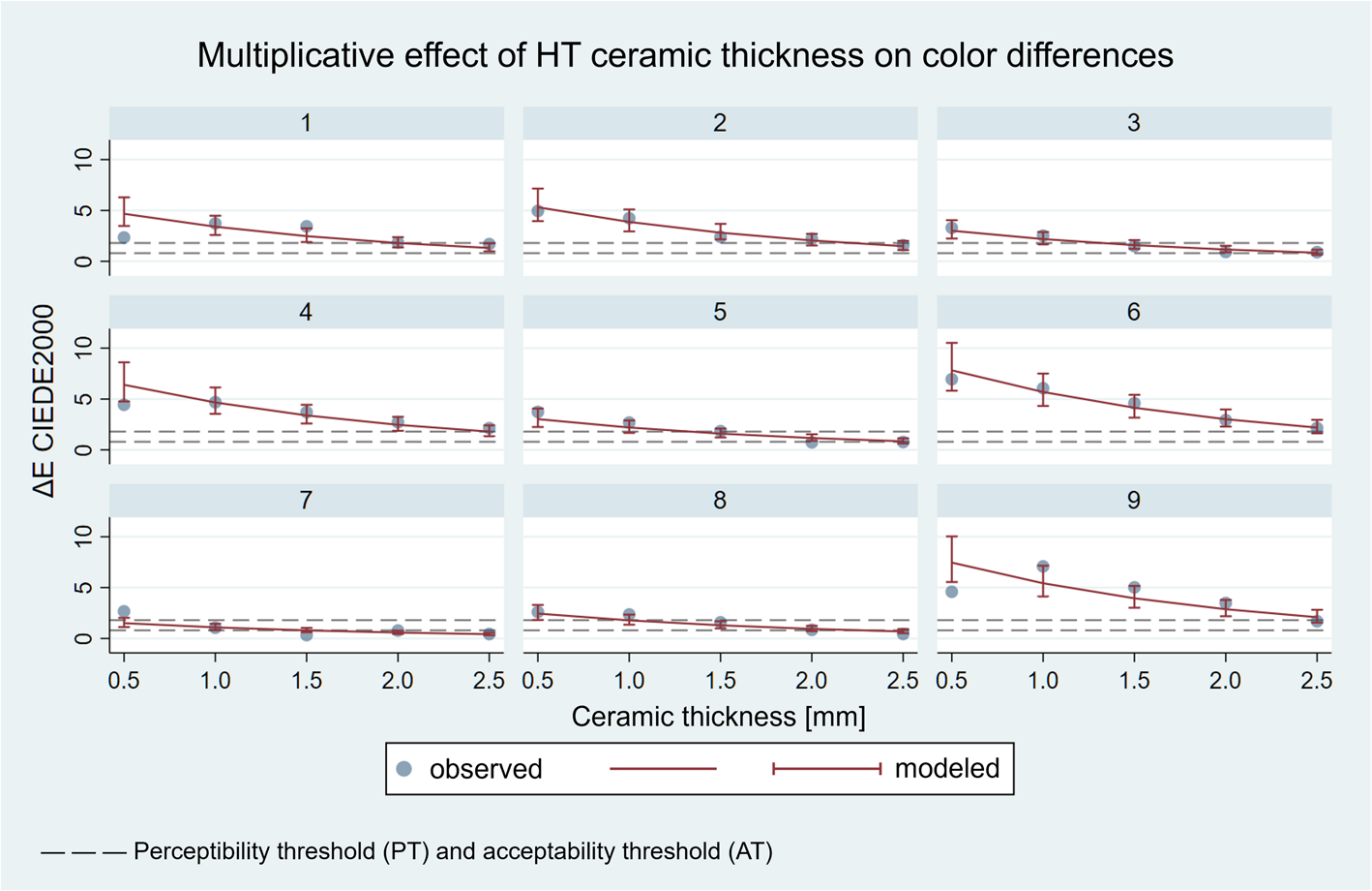
Multiplicative effect of ceramic thickness on ΔE_00_ values of HT layered specimens modelled by linear regression analysis (*R*^2^ = 0.8689). Each panel corresponds to the measurements of the indicated substrate

**Fig. 7:**
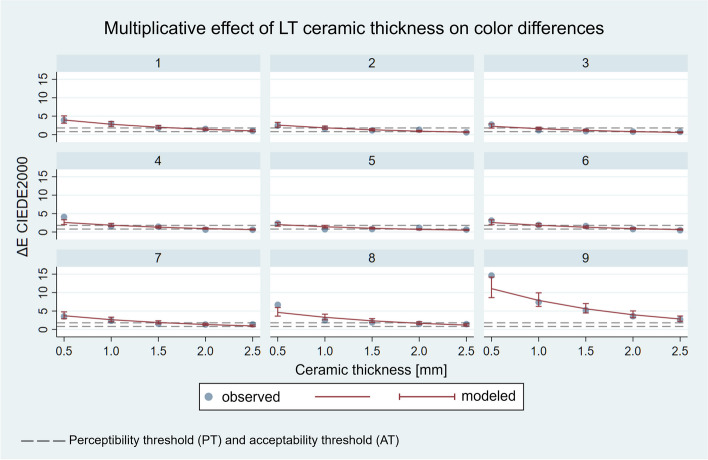
Multiplicative effect of ceramic thickness on ΔE_00_ values of LT layered specimens modelled by linear regression analysis (*R*^2^ = 0.9021). Each panel corresponds to the measurements of the indicated substrate

The results of the linear regression are shown in Figs. 6 and 7. The 9 panels detail the results for the 9 substrates.

Multiplicative effect analysis revealed that in the examined range of 0.5–2.5 mm thickness, ΔE_00_ changes according to a constant multiplier as the thickness increases. In the case of the HT specimens, by increasing the thickness by 0.5 mm, *ceteris paribus*, ΔE_00_ decreases to 0.728 times the initial value – in other words, to 72.8% of the initial value – according to the model. This relationship exists for all the samples the layer thicknesses of which differ by 0.5 mm. The effect is highly significant (*p* < 0.0001), and its 95% confidence interval ranges from 0.683 to 0.775. By increasing the thickness difference, ΔE_00_ decreases exponentially, e.g., an increase of 1.5 mm in thickness reduces the ΔE_00_ value by 0.728 [3] times. In the case of the LT specimens, an increase in thickness of 0.5 mm reduces the ΔE_00_ value to 71.1%, *p* < 0.0001, and its 95% confidence interval ranges from 0.674 to 0.750.

**Fig. 8:**
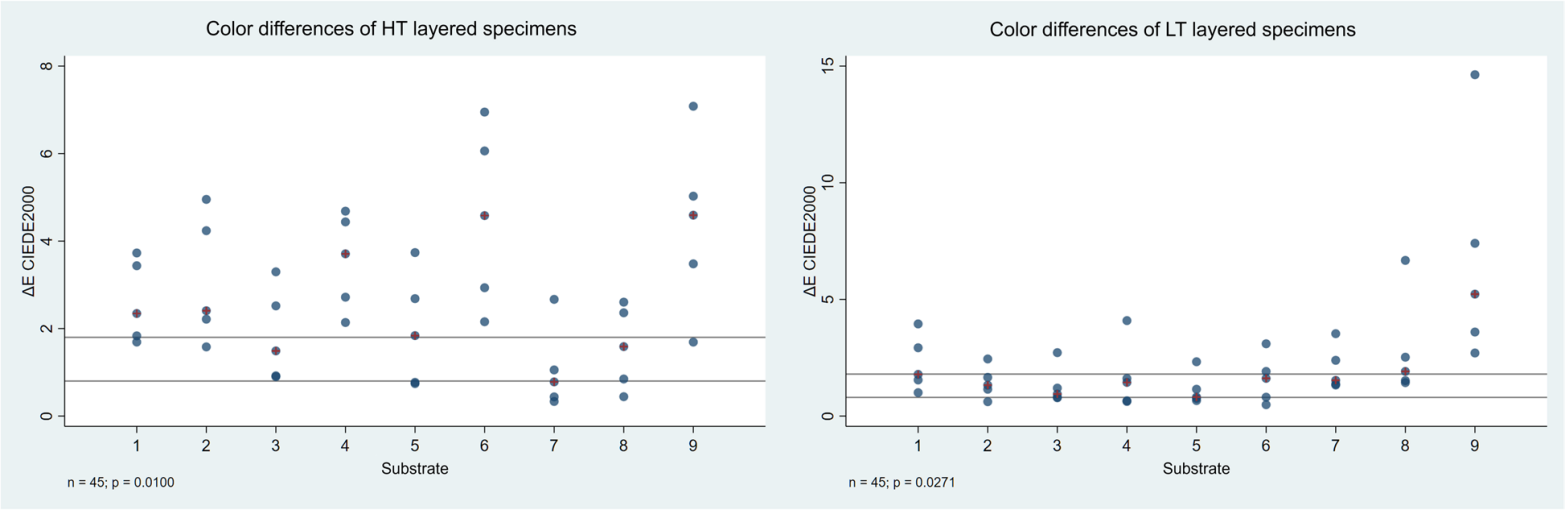
Dependence of ΔE_00_ values of the HT and LT layered specimens on the substrate. Reference samples: HT and LT blocks. The median of each group is indicated by a red cross. PT_50:50%_ = 0.8 and AT_50:50%_ = 1.8 are marked on the diagram

The relationship between the colour difference (ΔE_00_) of the HT and LT layered specimens from the reference and substrate materials is shown in Fig. 8. Beside every substrate (1-9), there is a group of 5 observations corresponding to the measurements with the 5 different ceramic thicknesses. The median of each group is indicated by a red cross. Perceptibility (PT_50:50%_ = 0.8) and acceptability (AT_50:50%_ = 1.8) thresholds are marked by horizontal lines.

**Fig. 9:**
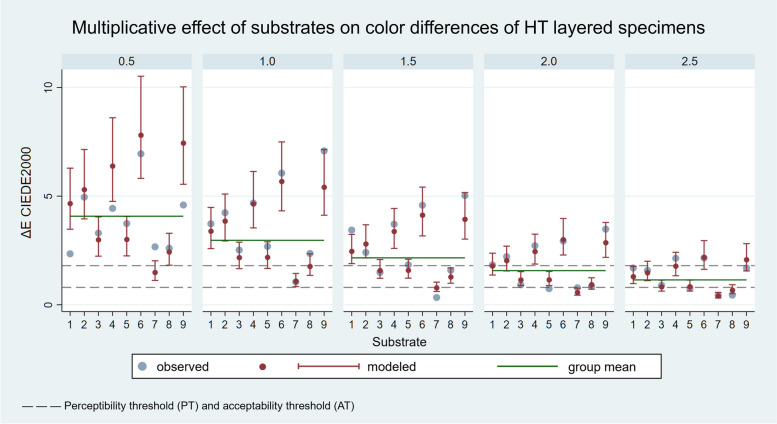
Multiplicative effect of the substrate on ΔE_00_ values of HT layered specimens modelled by linear regression analysis (*R* ^2^ = 0.8689). Each panel corresponds to the measurements of the indicated ceramic thickness. Group means are indicated by green lines

**Fig. 10:**
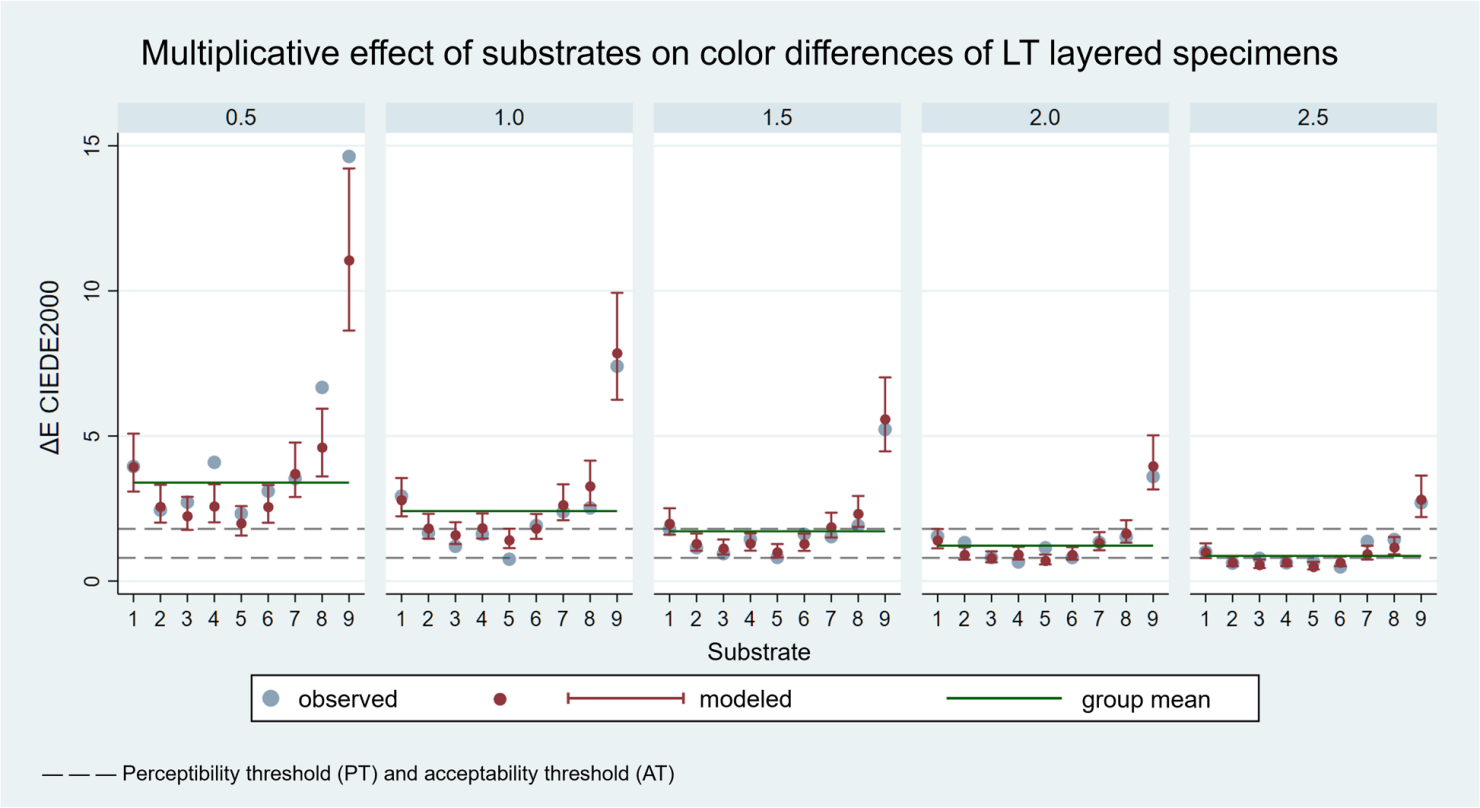
Multiplicative effect of the substrate on ΔE_00_ values of LT layered specimens modelled by linear regression analysis (*R*^2^ = 0.9021). Each panel corresponds to the measurements of the indicated ceramic thickness. Group means are indicated by green lines

The results of the linear regression are shown in Figs. 9 and 10. The 5 panels detail the results for the 5 ceramic thicknesses.

The effect of the substrate can be modelled with a constant multiplier as a characteristic of the material, which estimates the relationship between the ΔE_00_ mean measured in the given ceramic thickness group and the ΔE_00_ values of the layered specimens. The group mean within the panels (green line) varies depending on the ceramic thickness. The effect can be modelled, is independent of the layer thickness, and is constant. In the diagrams, the positive or negative deviation from the mean value is significant (*p* < 0.05) if the modelled confidence intervals marked in red do not intersect with the green line representing the group mean.

The effect of translucency was evaluated by comparing the results of the HT and LT materials. During the analysis of the effect of ceramic thickness, it was found that with an increase in thickness of 0.5 mm, the ΔE_00_ value decreases to 72.8% in the case of the HT ceramic and to 71.1% in the case of the LT ceramic. Figure 5 illustrates the relationship between the ΔE_00_ values of the HT and LT layered specimens and the layer thickness, and the group medians show different values for the two materials. In the case of the HT ceramic, up to a layer thickness of 2.0 mm, the group median exceeds the acceptability threshold; only at a thickness of 2.5 mm is it below. Beside the LT ceramic, the group median is already in the acceptable range at a layer thickness of 1.5 mm, and at a thickness of 2.5 mm, it is below the perceptibility threshold.

## Discussion

The reflectance spectra of the HT and LT ceramic blocks show the same characteristics, but the reflectance of the LT ceramic is higher in the entire investigated wavelength range. The difference increases in parallel with the wavelength, and the correlation is linear. According to the spectra, the HT ceramics reflect less light in the entire visible spectrum than the LT ceramics, i.e., the absorption and transmission of light through the material are greater. This effect is most pronounced in the near-red region of the spectrum. The different translucencies of the HT and LT ceramics and, consequently, the different reflectance spectra are caused by the diverse sizes and amounts of lithium disilicate crystals in the materials [17, 23]. HT ceramics contain fewer and larger crystals (1.5 × 0.8 μm), while LT ceramics contain more but smaller crystals (0.8 × 0.2 μm) [16].

The effect of an increase in ceramic thickness is reflected in the consistent decrease in group medians, i.e., the ΔE_00_ values of the HT and LT samples decrease. Linear regression analysis provides a quantitative characterization of the influencing effect of the layer thickness, which also appears to be a constant multiplier in the case of HT and LT ceramics. The results fit the model well, and the effect was significant (*p* < 0.0001); in light of this, the first null hypothesis was rejected. In the case of the HT ceramics with a thickness of 0.5 mm, the ΔE_00_ value of not even one sample was below the acceptability threshold, while with a layer thickness of 2.5 mm, 7 samples gave acceptable results, 3 of which were below the perceptibility threshold. In the case of LT ceramics, a thickness of 0.5 mm also did not yield acceptable results; however, with a ceramic thickness of 2.5 mm, the colour difference of 8 samples became acceptable, 5 of which were below the perceptibility threshold. Our results are in agreement with the results of previous studies regarding the effect of thickness [18, 22, 23, 25–27, 30, 31]. The effects of ceramic thickness, translucency and cement colour on the masking capacity of lithium disilicate glass-ceramics were investigated by Pala et al. on substrates made of bovine dentin stained with black tea [22]. In this study, human evaluators, rather than spectrophotometrical devices, were used to assess colour differences. According to the research, the masking ability is significantly influenced by the layer thickness of the ceramic, which depends on the translucency and cement colour. Ceramics with a thickness of 0.4–0.6 mm covered the examined colour difference [22]. During the spectrophotometric examination of lithium disilicate crowns, Czigola et al. found that although ceramic thickness has an effect on masking capacity, its role is limited in the case of HT ceramics [23]. According to Fachinetto et al., the ceramic thickness of lithium disilicate glass-ceramics has a significant impact on the ΔE_00_ values [30].

The effect of different substrate colours strongly influence the colour reproduction ability of the ceramic. According to the linear regression results, in the case of the HT ceramics, ND1 and ND2 substrates had no significant effect on the ΔE_00_ values compared to the group mean. The results for the specimens with ND4, ND6 and ND9 substrates were significantly worse, and those with ND3, ND5, ND7 and ND8 substrates were significantly better than the group mean (*p* < 0.05). In the case of the LT ceramics, the results for ND1 and ND7 substrates were not significantly different from the mean. The results for the specimens with ND8 and ND9 substrates were significantly worse, and those with ND2, ND3, ND4, ND5 and ND6 substrates were significantly better than the group mean (*p* < 0.05). Therefore, substrate colour significantly influences the colour reproduction ability of lithium disilicate glass-ceramics in several cases, the second null hypothesis was partially rejected. The reason for different results of the HT and LT ceramics is to be found in different levels of translucency. HT samples have a higher transmission of the substrate colour, therefore its effect on the ΔE_00_ value of the entire layered sample is greater. Correspondingly, within the groups according to layer thicknesses, standard deviation of ΔE_00_ values is larger, thus also the deviation of the individual samples from the group mean. In the case of the LT samples, where the standard deviation of ΔE_00_ values within the groups is smaller due to the lower translucency, the outliers have a greater influence on the group mean, so significant differences are obtained in the case of several samples. The most severely discoloured ND9 substrate gave significantly worse results than the group mean for both ceramic types. The effect of substrate or background colour has also been discussed in previous studies, although mainly with fewer substrate samples [1, 18, 21, 24–27, 29]. Comba et al. evaluated the effects of substrate and cement shades on the translucency and colour of lithium disilicate and zirconia CAD/CAM materials [21]. The results showed that background shade significantly influenced the translucency and colour of the tested ceramic materials. Moreover, the final colour of high translucency lithium disilicate restorations is mainly affected by the shade of the core material [21]. Czigola et al. used 12 different substrates to evaluate the effects of substrate colour, ceramic thickness and translucency, and cement shade on the colour difference from a reference colour of lithium disilicate crowns [23]. The study revealed that there was no combination under the AT_50:50%_ (ΔE_00_ = 1.8) with gold alloy substrates, and only one combination was below the AT_50:50%_ with Co-Cr substrates (1.5 mm LT crown, light plus try-in paste). The lowest ΔE_00_ values were found for LT 1.5 mm thick crowns [23]. Sancaktar et al. investigated the effect of ceramic thickness, background and cement shade on the translucency of zirconia reinforced lithium silicate and lithium disilicate ceramics [26]. In this study, although low translucent ceramic materials were used, the background colour affected the final translucency [26].

The different behaviours of the HT and LT ceramics can be seen in several results. The comparison of the reflectance spectra of the two types of materials showed that closer to the red regions of the visible spectrum, the reflectance of the HT ceramic is lower, i.e., the reflected light contains fewer yellowish-reddish components than in the case of the LT ceramics. This can result in a grayer, cooler shade of the high translucency material. As mentioned above, the colour reproduction ability of the two materials is affected differently by the increase in layer thickness, which is reflected in the different multipliers. The location of the group medians calculated for each ceramic thickness also indicates the different optical behaviours of the two translucencies. Although a thickness of 0.5 mm did not yield acceptable results in the case of any ceramic, a thickness of 1.0 mm produced acceptable results in the case of the HT ceramic with 1 substrate and in the case of the LT ceramic with 4 substrates, one of which was even below the perceptibility threshold. Based on the aforementioned results, as translucency definitely has a significant effect on the colour reproduction ability of lithium disilicate glass-ceramics, the third null hypothesis was also rejected. Our results are in agreement with the results of previous studies regarding the effect of translucency [22–24, 27, 29, 30, 32]. Skyllouriotis et al. determined the translucency of 6 materials used for veneer restorations by assessing their translucency parameters, contrast ratios, and potential to mask dark tooth colours [32]. It was found that low translucency lithium disilicate materials appeared to be opaquer than the other ceramics tested and therefore have better masking properties [32].

To the best of the authors’ knowledge, the optical properties of lithium disilicate glass-ceramics have not been investigated so comprehensively before. The present study determined the colour reproduction ability of ceramics with 2 different translucencies and 5 layer thicknesses on 9 substrate colours. Although some of the results of this study are in accordance with previous research results, it contains new findings that carry important information for clinical practice and can provide guidelines for choosing the right material with the appropriate translucency and layer thickness.

It is important to emphasize that the results come from in vitro research which need to be taken with caution outside of the experimental environment, although the method used is adequate for examining factors influencing the colour reproduction ability of lithium disilicate glass-ceramic materials.

## Conclusions

Within the limitations of the present study, the following conclusions were established:

- The layer thickness of lithium disilicate glass-ceramics significantly affects the colour reproduction ability. A thickness of 0.5 mm is not sufficient to achieve an acceptable result at any level of translucency, while the low translucency ceramic at a thickness of 1.5 mm provides acceptable results, except for severely discoloured substrates (ND8 and ND9). The colour reproduction ability of the examined lithium disilicate glass-ceramics increases exponentially with increasing layer thickness, according to a constant multiplier, as a characteristic of the material.
- Based on the examination of 9 substrate materials, it was found that in the case of high and low translucency lithium disilicate glass-ceramics, 7 substrate materials significantly affect the colour reproduction ability of the ceramics.
- The colour reproduction abilities of high and low translucency lithium disilicate glass-ceramics are significantly different.

## Data Availability

All data generated and analysed during this study are included in this published article as figures. The datasets generated and analysed during the current study are available from the corresponding author on reasonable request.

## Abbreviations

AT: Acceptability threshold
CAD/CAM: Computer aided design/computer aided manufacturing
CIE: Commission Internationale de l’Éclairage
HT: High translucency
I: Impulse
LS_2_: Lithium disilicate glass-ceramic
LT: Low translucency
MO: Medium opacity
MT: Medium translucency
PT: Perceptibility threshold
SCI: Specular component included
